# Impact of hyperuricemia and chronic kidney disease on the prevalence and mortality of cardiovascular disease in cancer survivors

**DOI:** 10.1002/cam4.7180

**Published:** 2024-04-30

**Authors:** Yanlin Chen, Yuhan Chen, Weidong Lin, Lu Fu, Huiyi Liu, Sijia Pu, Haowei Chen, Hong Yi, Yumei Xue

**Affiliations:** ^1^ Department of Guangdong Cardiovascular Institute, Guangdong Provincial People's Hospital (Guangdong Academy of Medical Sciences) Southern Medical University Guangzhou China; ^2^ School of Medicine South China University of Technology Guangzhou China; ^3^ The Second School of Clinical Medicine Southern Medical University Guangzhou China

**Keywords:** cancer survivors, cardiovascular diseases, hyperuricemia, mortality, soluble uric acid

## Abstract

**Background:**

The risks of cardiovascular disease (CVD) and CVD mortality are prevalent among cancer survivors (CS) population. The 2022 ESC Guidelines on cardio‐oncology have recommended that modifying cardiovascular risk factors (CVRF) could potentially improve long‐term outcomes in CS.

**Objectives:**

To identify the independent and joint chronic kidney disease (CKD) associations of hyperuricemia with the incidence of CVD and mortality outcomes among CS.

**Methods:**

Utilizing data from the US National Health and Nutrition Examination Survey spanning 2005–2018, we assessed the risk of CVD through weighted multivariable logistic regression and restricted cubic spline (RCS) regression. Additionally, all‐cause and CVD‐related mortality were evaluated using weighted multivariable Cox regression and Kaplan–Meier analysis. Subgroup analysis was conducted to further elucidate the interplay between hyperuricemia, CKD, and mortality within the CS population.

**Results:**

A total of 3276 CS participants were enrolled in this study. Results showed that hyperuricemia was positively related to the incidence of CVD (OR [95% CI] = 1.86 [1.24, 2.81], *p* = 0.004). RCS analysis further demonstrated that uric acid levels ≥345 μmol/L positively correlated with CVD incidence (*p* value for nonlinearity = 0.0013). However, the association between hyperuricemia and CVD mortality, as well as all‐cause mortality did not reach statistical significance in the fully adjusted model (HR = 1.48, 95% CI: 0.92–2.39, *p* = 0.11; HR = 1.11, 95% CI:0.92, 1.34, *p* = 0.28, respectively). Among CS participants with CKD, hyperuricemia could increase risks of all‐cause (HR [95% CI] = 1.39 [1.08, 1.11], *p* = 0.02) and CVD mortality (HR [95% CI] =2.17 [1.29, 3.66], *p* = 0.004) after adjusting for sex, age, and ethnicity.

**Conclusions:**

In the CS population, hyperuricemia was positively associated with the incidence of CVD. In addition, CKD might be an intermediate variable among the CS population that mediated the effects of hyperuricemia on mortality.

## 1 INTRODUCTION

Cancer and cardiovascular diseases (CVDs) are the most prevalent chronic diseases in the United States (US). By 2019, it is estimated that more than 16.9 million cancer survivors (CSs) in the US, and this number is continuously growing to more than 22.1 million until 2030 due to early cancer screening and treatment.^1^ Existing study shows that CSs have the highest risk of cardiovascular complications in the first year after their cancer diagnosis.^2^ This heightened risk of CVD and CVD‐related mortality, compared to the general population, is partly attributed to the cardiotoxic effects of cancer therapy.^3^ In addition, recent guidelines from the *European Society of Cardiology* (*ESC* 2022) have recommended that a growing understanding and modification of cardiovascular risk factors (CVRF, including dyslipidemia, metabolic syndrome, diabetes, and obesity) may improve long‐term outcomes in CS, especially responsible for CVD mortality.^3^ Despite these recommendations, the identification and treatment of CVRF remain insufficiently addressed and diagnosed within the CS population, warranting increased attention to other potential risk factors.

Uric acid as the final product of purine is associated with various health issues. Soluble uric acid (SUA) elevation has been defined as hyperuricemia. In 1951, Gertler et al. first reported that a high level of SUA could increase the risk of myocardial infarction.^4^ Since then, further clinical evidence has shown a positive relationship between high SUA levels and CVD and CVD mortality.^5^, ^6^, ^7^ Moreover, a prospective cohort study found that SUA was elevated after surgery for renal tumors,^8^ suggesting SUA elevation as a risk factor for CVD and a potential contributor to cancer‐related complications. Despite these findings, limited evidence exists regarding the relationship between SUA and CVD, as well as CVD‐related mortality within the CS population. Notably, a study highlighted the influence of glomerular filtration rate (GFR) on SUA levels, indicating a positive correlation between creatinine clearance and 24‐h UA excretion.^9^ Therefore, we used the National Health and Nutrition Examination Survey (NHANES) dataset to explore the potential association between SUA levels among the CS population and the incidence of cardiovascular events. Furthermore, we also try to discuss the role of GFR in CS patients with hyperuricemia.

## 2 METHODS

### 2.1 Data collection

This cross‐sectional and longitudinal study used data from the 2005–2018 cycles of NHANES, a large clinical database, representing the US civilian non‐institutionalized population. The NHANES database includes six parts: demographics data, dietary data, examination data, laboratory data, questionnaire data, and limited access data. However, for this study, we mainly used demographics, laboratory, and questionnaire data. The health‐related information collected is supported by the Centers for Disease Control (CDC) and Prevention's National Center for Health Statistics (NCHS). This dataset is ethically approved by the NCHS Research Ethics Review Board, and written informed consent is obtained from all participants or their proxies.

### 2.2 Study design

**FIGURE 1:**
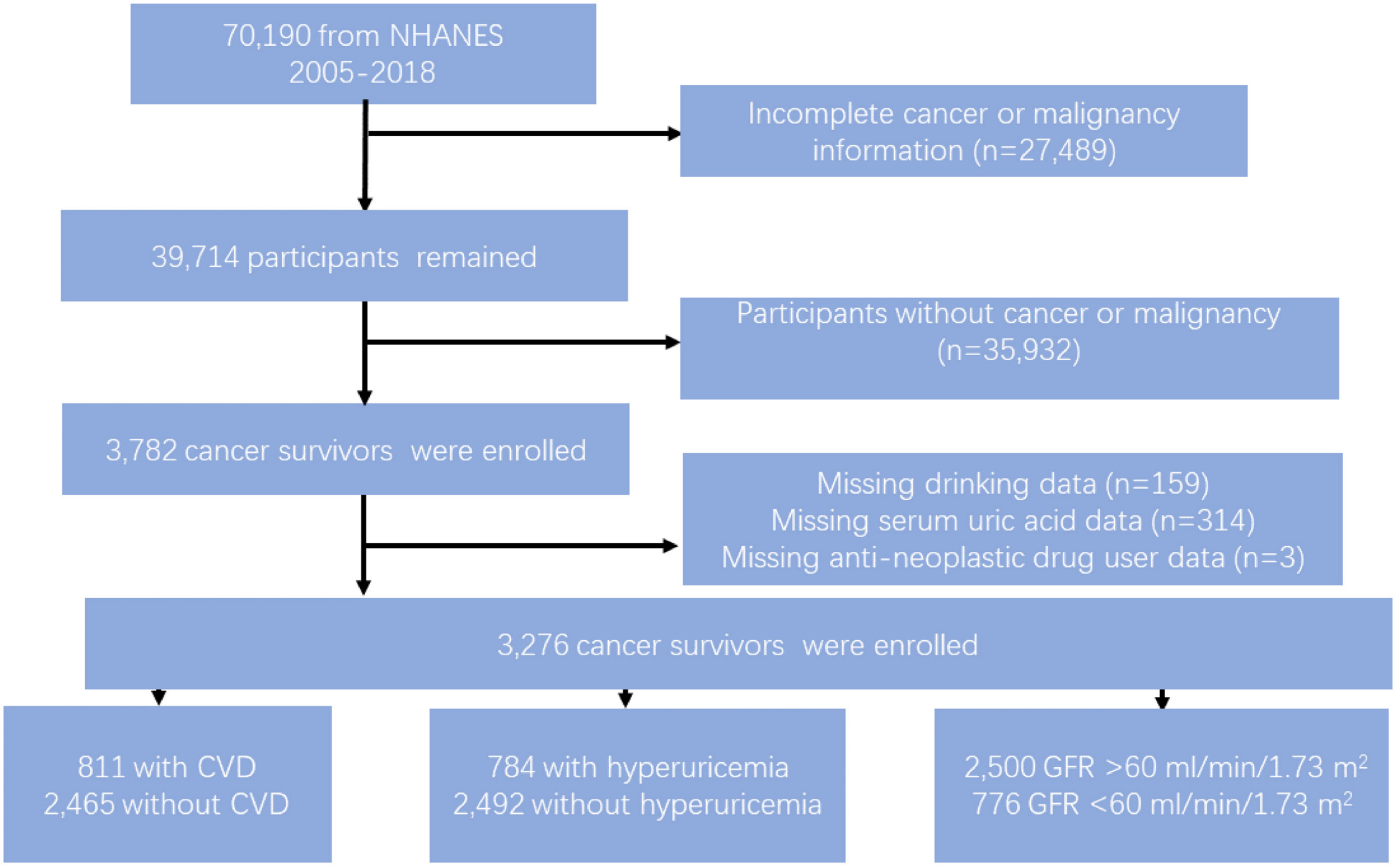
The flowchart of study sample selection form NHANES 2005–2018.

Study mainly focused on the CS population from the 2005–2018 NHANES cycles (shown in Figure 1). Participants were asked the following question: “Have you ever been told by a doctor or other health professional that you had cancer or malignancy of any kind?” Participants were enrolled as CS if they responded yes. For cancer types, these enrolled participants would further ask “What kind of cancer was it?” Next, the exposure variables were selected, including SUA (μmol/L) and GFR. The SUA levels could be obtained directly from laboratory parts of NHANES, and the primary definition of hyperuricemia was set as SUA >420 μmol/L for males and a SUA >360 μmol/L for females.^10^ GFR was calculated by the *Chronic Kidney Disease Epidemiology Collaboration* (*CKD‐EPI*) equation (mL/min/1.732 m^2^)^11^ According to the 2004 international guideline from the Kidney Disease Improving Global Outcomes (KDIGO),^12^ the GFR < 60 mL/min/1.732 m^2^ for the definition of CKD. This cutoff value of 60 mL/min/1.732 m^2^ was utilized in the study to transform the GFR levels into categorical variables.

The outcomes of interest include the prevalence of CVD, all‐cause mortality, and CVD‐specific mortality. Participants were asked the following questions: “Has a doctor or other health professional ever told you that you have coronary heart disease/angina/congestive heart failure/heart attack/stroke?” Participants were recognized as CVD patients if they responded yes. Otherwise, mortality data were obtained from “2019 Public‐Use Linked Mortality Files”, which were matched with the National Death Index (NDI) (https://www.cdc.gov/nchs/data‐linkage/mortality‐public.htm), and the mortality data was recorded up to December 31, 2019.

### 2.3 Covariant evaluation

The covariates are mainly divided into sociodemographic variables (age, sex, ethnicity, poverty status, education level, and body mass index), lifestyle variables (smoking, alcohol user), and diseases relevant to the outcomes (hypertension, diabetes and antineoplastics). Education level was grouped into <12 years and ≥ 12 years. Poverty level was evaluated by poverty income ratio (PIR), which was classified into three groups: low income <1, 1 ≤ middle income<3, and high income≥3. Body mass index (BMI) was calculated as weight (kg)/(height (m))^2^, which was classified into three groups: <25, 25–30, and ≥ 30 (kg/m^2^). Hypertension was defined as a physician's diagnosis of hypertension or self‐reported taking anti‐hypertensive drugs, or the blood pressure ≥ 140/90 mmHg. Diabetes was defined by self‐reported diagnosis, fasting plasma glucose≥7 mmol/L, 2‐h plasma glucose≥11.1 mmol/L, glycated hemoglobin A_1c_ ≥ 6.5%, or currently taking antihyperglycemic agents. Information on antineoplastics use was gathered from a household interview. Participants were asked: “have you taken antineoplastics in the past?” Participants who responded “yes” would be asked to show the containers of antineoplastics. If unavailable, the CS participants were asked to provide the names of the antineoplastics. For each name reported, the interviewer recorded the antineoplastics to match the Lexicon Plus, a database that includes all prescription drugs. The antineoplastics including alkylating derivatives, antimetabolites, antineoplastic hormones, VEGF/VEGFR inhibitors, BCR‐ABL tyrosine kinase inhibitors, HER2 inhibitors, and so forth. (more details could refer to the following website: https://www.cerner.com/solutions/drug‐database).

### 2.4 Statistical analysis

According to the NHANES guidelines, appropriate weighting of complex survey data is needed to properly represent the US population. Therefore, the calculated formulate for 2005–2018 circles is as follows: (1/7) × WTMEC2YR_2005–2018_, where WTMEC2YR represents the weight variable for 2005–2018. Continuous variables were presented as means ± standard error (SE), while categorical variables were presented as frequency with percentage. Statistical methods, including *t*‐tests, Mann–Whitney *U*‐tests, ANOVA and Chi‐squared tests, were applied to compare the differences in baseline data across the prevalence of CVD. Weighted logistic regression was performed to explore the association between hyperuricemia and the prevalence of CVD in the CS population, the results were represented by odds ratios (OR) with 95% confidence intervals (CI). Furthermore, weighted restricted cubic spline (RCS) regression was conducted to explore the non‐linear relationship between hyperuricemia and CVD in the CS population. Weighted Cox regression analysis was performed to estimate the hazard ratios (HR) and 95% CI for all‐cause and CVD mortality affected by hyperuricemia. Kaplan–Meier (K–M) analysis was also performed to evaluate the all‐cause and CVD mortality. In addition, as around 70% of SUA is expelled from the kidneys, elevated SUA levels were associated with kidney function decline.^13^ Subgroup analyses were further conducted. CS participants were divided into normal renal function (GFR >60 mL/min/1.732 m^2^) and CKD groups (GFR ≤60 mL/min/1.732 m^2^) to verify whether increased CVD and CVD mortality in CS patients result from elevated SUA levels or from SUA‐induced kidney damage. To remove the covariates effect, three analysis models have been established: model 1 without covariate adjustment, model 2 adjusted for sex, age, and ethnicity, and model 3 was the fully adjusted model. All analyses were conducted using R software (version 4.3.0), and *p* value <0.05 was considered statistically significant.

## 3 RESULTS

### 3.1 Baseline characteristics of study participants

Study data was collected from the 2005–2018 cycles of NHANES. A total of 70,190 subjects were screened. Among them, we initially enrolled 39,714 participants who had cancer or malignancy. After excluding individuals with missing data on SUA levels, alcohol consumption, antineoplastics use, and those lacking information on CVD and diabetes, a final cohort of 3276 CS participants was included for further analysis (shown in Figure 1). These CS participants represented approximately 20,796,857 noninstitutionalized residents in the US. The number of CS was classified by cancer types and survival status, as detailed in Table S1 of the supplement. The baseline characteristics were shown in Table 1. In summary, the average age of the participants was 63 years. Among them, 811 (25%) had CVD, 784 (24%) had hyperuricemia, 2500 (76%) had CKD, and 184 (6%) of them died due to CVD. In the group with hyperuricemia, most of them were male, older, non‐Hispanic White, with high PIR values, and most of them had a higher BMI and CKD history. According to baseline data analysis, no statistically significant differences were observed in antineoplastics use and different neoplastic types between two groups (shown in Table 1).

**TABLE 1:** The baseline characteristics of the CS in NHANESE 2005–2018, statistical analysis was presented with mean ± SD and percentage (frequency).

Variable	Total (3276)	Non‐hyperuricemia (2492)	Hyperuricemia (784)	*p* value
Age	62.70 ± 0.35	60.88 ± 0.41	70.14 ± 0.53	**<0.0001**
Sex				
Female	1732 (52.82)	1386 (59.25)	346 (47.56)	0.84
Male	1547 (47.18)	1081 (40.75)	466 (52.44)	
Ethnicity				
Non‐Hispanic White	2248 (68.56)	1652 (86.57)	596 (85.20)	**<0.0001**
Non‐Hispanic Black	450 (13.72)	334 (4.67)	116 (6.14)	
Mexican American	222 (6.77)	181 (2.66)	41 (2.02)	
Other	359 (10.95)	300 (6.09)	59 (6.64)	
Education				
<12	1426 (43.53)	1006 (30.84)	420 (45.50)	0.09
≥12	1850 (56.47)	1459 (69.16)	391 (54.50)	
Poverty level				
Low income	741 (24.67)	513 (13.74)	228 (20.91)	**<0.0001**
Middle income	1211 (40.31)	867 (32.83)	344 (47.60)	
High income	1052 (35.02)	872 (53.43)	180 (31.49)	
Smoke				
No	2759 (84.22)	2089 (84.63)	670 (81.98)	**0.02**
Yes	517 (15.78)	375 (15.37)	142 (18.02)	
Alcohol user				
No	1051 (35.82)	734 (25.58)	317 (39.10)	**<0.001**
Yes	1883 (64.18)	1484 (74.42)	399 (60.90)	
Diabetes				
No	791 (90.4)	481 (92.71)	310 (88.22)	**0.002**
Yes	84 (9.6)	37 (7.29)	47 (11.78)	
Hypertension				
No	761 (86.97)	455 (88.41)	306 (86.38)	0.08
Yes	114 (13.03)	63 (11.59)	51 (13.62)	
BMI				
<25	884 (27.5)	707 (29.67)	177 (22.68)	**<0.0001**
25–30	1124 (34.97)	837 (34.86)	287 (34.81)	
≥30	1206 (37.52)	885 (35.47)	321 (42.51)	
CKD				
No	777 (23.7)	448 (14.06)	329 (37.41)	**<0.0001**
Yes	2502 (76.3)	2019 (85.94)	483 (62.59)	
Hyperuricemia				
No	2494 (76.06)	1962 (81.44)	532 (68.56)	**<0.0001**
Yes	785 (23.94)	505 (18.56)	280 (31.44)	
Antineoplastics use				
No	3068 (93.65)	2342 (94.42)	726 (92.92)	0.28
Yes	208 (6.35)	150 (5.58)	58 (7.08)	
Neoplastic types				
Gastrointestinal neoplasm	287 (8.76)	210 (6.35)	77 (7.39)	0.76
Endocrine system tumor	570 (17.4)	418 (17.27)	152 (18.48)	
Genitourinary system tumors	1080 (32.97)	832 (26.77)	248 (26.94)	
Respiratory system neoplasm	82 (2.5)	58 (1.87)	24 (2.31)	
Circulatory system neoplasm	107 (3.27)	88 (3.58)	19 (1.87)	
Neurological neoplasms	15 (0.46)	13 (0.32)	2 (0.23)	
Melanoma	199 (6.07)	147 (7.85)	52 (7.49)	
Skin (non‐melanoma)	519 (15.84)	399 (21.44)	120 (21.63)	
Skin (don't know what kind)	245 (7.48)	197 (8.93)	48 (7.67)	
Soft tissue (muscle or fat)	5 (0.15)	4 (0.12)	1 (0.34)	
Bone	10 (0.31)	8 (0.27)	2 (0.18)	
Other	157 (4.79)	118 (5.21)	39 (5.47)	

Abbreviations: BMI, body mass index; CKD, chronic kidney disease; CVD, cardiovascular disease.

*Note*: Bold values indicate statistically significance of *p* < 0.05.

### 3.2 Associations between hyperuricemia and CVD

**FIGURE 2:**
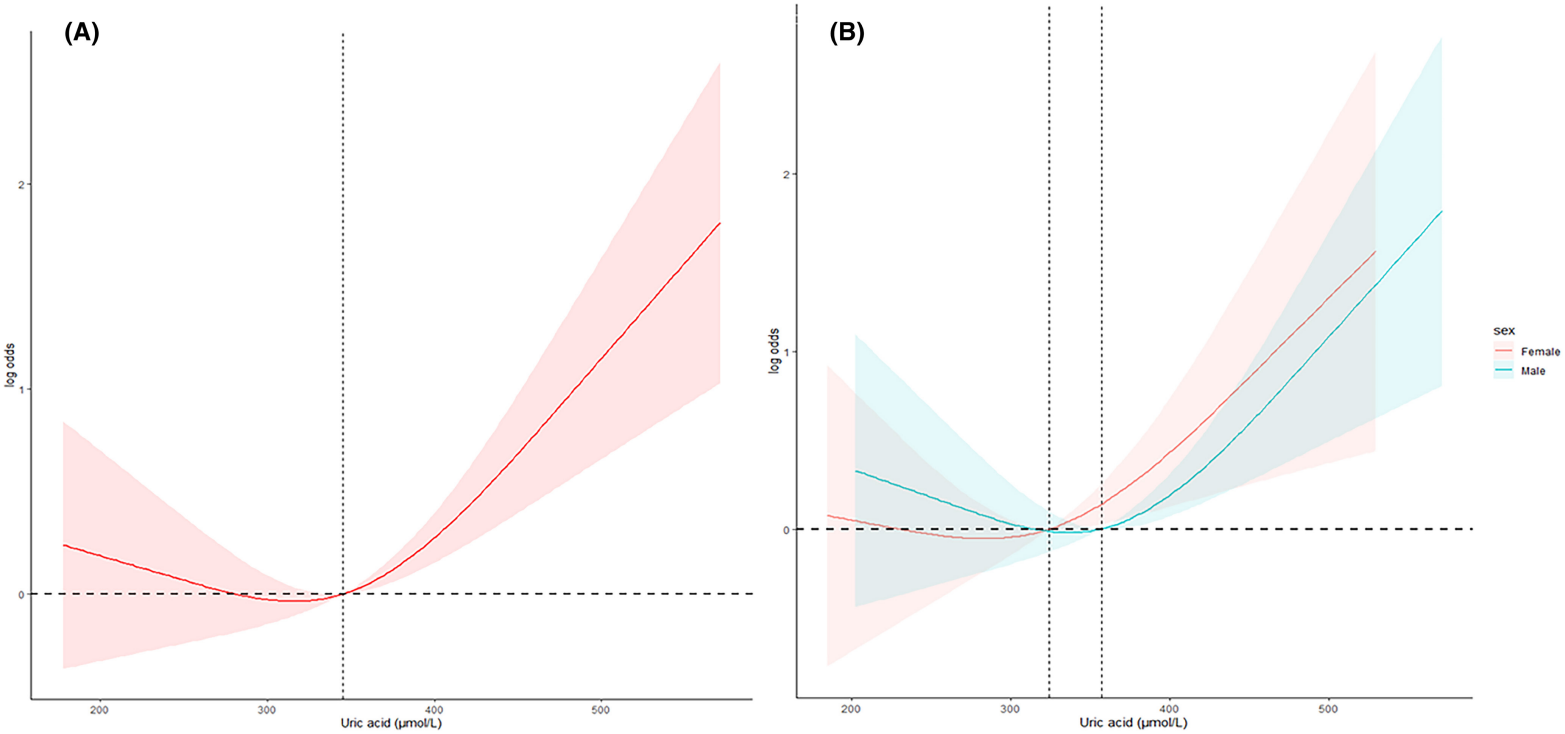
Weighted RCS logistic analysis on the association between the SUA levels and the risk of CVD. (A) RCS curve of the association between the SUA levels and CVD among all the CS participants, reference median of SUA was 345 μmol/L; (B) RCS cure of the association between the SUA levels and CVD among male and female CS participants, reference median for female was 324.2 μmol/L, reference median for male was 356.9 μmol/L. All of potential covariates were adjusted in the RCS. CS, cancer survivor; CVD, cardiovascular disease; RCS, restricted cubic spline; SUA, soluble uric acid.

Weighted logistic regression analysis indicated a significant association between hyperuricemia and the development of CVD in CS individuals. Hyperuricemia was positively related to odds ratio (OR) of CVD across all three models (Model 1: OR [95% CI] = 2.01 [1.64, 2.46], *p* < 0.0001; Model 2: OR [95% CI] = 1.72 [1.39, 2.13], *p* < 0.0001; Model 3: OR (95% CI) =1.86[1.24, 2.81], *p* = 0.004, shown in Table 2). Based on this finding, weighted RCS logistic analysis was performed, and adjusting for all potential confounding variables, to further explore the association between hyperuricemia and CVD in CS participants. The results of the RCS analysis also revealed a positive nonlinear relationship between hyperuricemia and CVD incidence (*p* value for nonlinear = 0.0013). The RCS plot indicated that the risk of CVD might increase with rising uric acid levels, particularly when the uric acid levels ≥345 μmol/L (Figure 2A). Conversely, the risk of CVD decreased when SUA levels were at the physiological range (Figure 2A). Subgroup analysis classified by sex was also carried out and revealed that there was a gender‐based difference in the association between the uric acid levels and the incidence of CVD. In Figure 2B, the risk of CVD increased obviously when the uric acid levels ≥356.9 μmol/L in males (*p* value for nonlinear = 0.0064), while no statistically significant association was observed in females (*p* value for nonlinear = 0.1334).

**TABLE 2:** Weighted logistic was used to explore the association between hyperuricemia and the incidence of CVD.

Outcomes	OR (95% CI)	*p* value
Hyperuricemia—CVD		
Model 1	2.01 (1.64, 2.46)	**<0.0001**
Model 2	1.72 (1.39, 2.13)	**<0.0001**
Model 3	1.86 (1.24, 2.81)	**0.004**

*Note*: Bold values indicate statistically significance of *p* < 0.05.

### 3.3 Associations between hyperuricemia and mortality in CS

**FIGURE 3:**
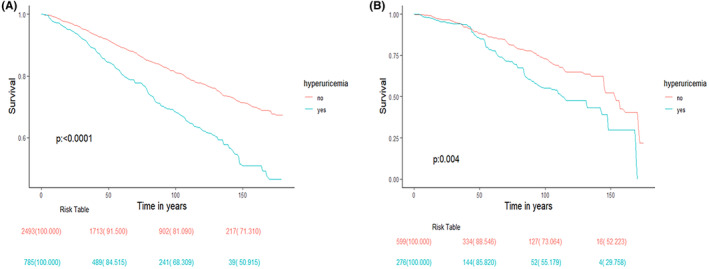
Kaplan–Meier curves depict the association between hyperuricemia and survival from 2005 to 2018 NHANES. (A) Kaplan–Meier curves the association between hyperuricemia and all‐cause of mortality among all the CS participants, (B) Kaplan–Meier curves the association between hyperuricemia and CVD mortality among all the CS participants. CS, cancer survivor; CVD, cardiovascular disease.

During 3279 person‐years of follow‐up (median follow‐up, 79.82 ± 1.46 months) from NHANES 2005–2018, a total of 874 (27%) deaths were documented, out of which 184 deaths (6%) were attributed to CVD. The results of weighted Cox regression analysis for all‐cause and CVD‐related mortality were presented in Table 3. According to model 1 and model 2, hyperuricemia was positively related to hazard ratio (HR) of all‐cause of mortality (Model 1: HR [95% CI] = 1.85 [1.55, 2.20], *p* < 0.0001; Model 2: HR [95% CI] = 1.41 [1.19, 1.66], *p* < 0.0001). While, no statistically significant increase in all‐cause of mortality was observed after full adjustment (Model 3: HR [95% CI] = 1.11 [0.92, 1.34], *p* = 0.28). Similarly, hyperuricemia was positively related to hazard ratio (HR) of CVD mortality (Model 1: HR [95% CI] = 1.66 [1.22, 2.25], *p* = 0.001; Model 2: HR [95% CI] = 1.69 (1.23, 2.33), *p* = 0.001). However, there was no statistical increase in CVD mortality after full adjustment (Model 3: HR [95% CI] = 1.48 [0.92, 2.39], *p* = 0.14). In the K–M study, CS participants were stratified with SUA levels (with or without hyperuricemia) and found that hyperuricemia was positively associated with an increase in both all‐cause and CVD mortality (*p* < 0.0001 and *p* = 0.004, respectively, shown in Figure 3).

**TABLE 3:** Cox regression models showing the association between hyperuricemia and all‐cause mortality/CVD mortality NHANES 2005–2018 (*N* = 3279).

Outcomes	HR (95% CI)	*p* value
Hyperuricemia—All‐cause of mortality		
Model 1	1.85 (1.55, 2.20)	**<0.0001**
Model 2	1.41 (1.19, 1.66)	**<0.0001**
Model 3	1.11 (0.92, 1.34)	0.28
Hyperuricemia—CVD mortality		
Model 1	1.66 (1.22, 2.25)	**0.001**
Model 2	1.69 (1.23, 2.33)	**0.001**
Model 3	1.48 (0.92, 2.39)	0.11

*Note*: Model 1: no covariate was adjusted. Model 2: adjusted for sex, age, and ethnicity. Model 3: fully adjusted sex, age, ethnicity, poverty level, smoke, alcohol user, diabetes, hypertension, BMI, CKD, antineoplastic drug user, and neoplastic type. Bold values indicate statistically significance of *p* < 0.05.

Abbreviations: BMI, body mass index; CKD, chronic kidney disease; CVD, cardiovascular disease; HR, hazard ratio; OR, odds ratio.

### 3.4 SUA and GFR relationship affect the association between hyperuricemia and mortality

**FIGURE 4:**
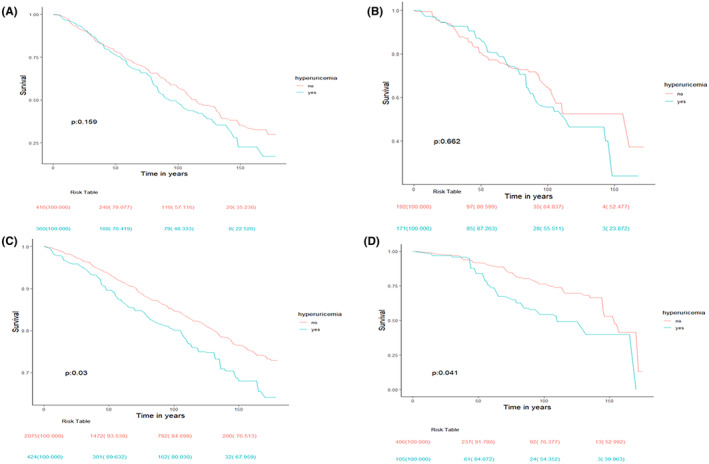
Kaplan–Meier curves depict the association between hyperuricemia and survival in CS participants with or without CKD. (A) Kaplan–Meier curve showed the association between hyperuricemia and all‐cause of mortality among no‐CKD CS participants, (B) Kaplan–Meier curve showed the association between hyperuricemia and CVD mortality among no‐CKD CS participants. (C) Kaplan–Meier curve showed the association between hyperuricemia and all‐cause of mortality among CS participants with CKD, (D) Kaplan–Meier curve showed the association between hyperuricemia and CVD mortality among CS participants with CKD. CKD, chronic kidney disease; CS, cancer survivor; CVD, cardiovascular disease.

Subsequently, we used weighted multivariate linear regression to explore the relationship between SUA and GFR in CS participants. We found that the SUA levels were negatively associated with GFR after fully adjusting potential covariates (*β* = −1.97, 95% CI: −2.42, −1.52, *p* < 0.0001, presented in Table S2 of the supplement). According to GFR, the CS participants were divided into CKD (*N* = 2500, CFR < 60 mL/min/1.732 m^2^) and no‐CKD (*N* = 776) groups. In the no‐CKD group, there was no statistically significant association between hyperuricemia and mortality (both all‐cause and CVD‐related mortality, shown in Table 4). However, in the CKD group, CS with hyperuricemia had higher all‐cause mortality (HR [95% CI] = 1.39 [1.08, 1.11], *p* = 0.02) as well as increased CVD mortality (HR [95% CI] = 2.17 [1.29, 3.66], *p* = 0.004) after adjusting age, ethnicity and sex, while, no statistically significant differences were observed after fully covariates adjusted, shown in Table 4. Meanwhile, the K–M study indicated that CS participants with CKD and hyperuricemia could increase the all‐cause and CVD mortality (*p* = 0.03 and *p* = 0.0411, respectively, as shown in Figure 4), while, no statistically significant differences were observed within the group without CKD (shown in Figure 4). The association between hyperuricemia and mortality could be influenced by the interplay between SUA and GFR.

**TABLE 4:** Weighted cox regression models showing the association between hyperuricemia and all‐cause mortality/CVD mortality in CS participants with or without CKD.

Outcomes	No‐CKD HR (95% CI)	*p* value	CKD HR (95% CI)	*p* value
Hyperuricemia—All‐cause of mortality				
Model 1	1.21 (0.93, 1.57)	0.16	1.43 (1.07, 1.90)	**0.02**
Model 2	1.13 (0.90, 1.42)	0.27	1.39 (1.08, 1.11)	**0.02**
Model 3	0.95 (0.75, 1.21)	0.67	1.17 (0.88, 1.56)	0.21
Hyperuricemia—CVD mortality				
Model 1	1.11 (0.70, 1.75)	0.66	2.06 (1.23, 3.43)	**0.01**
Model 2	1.21 (0.75, 1.95)	0.44	2.17 (1.29, 3.66)	**0.004**
Model 3	1.21 (0.60, 2.46)	0.59	1.07 (0.81, 3.56)	0.16

*Note*: Model 1: no covariate was adjusted. Model 2: adjusted for sex, age, and ethnicity. Model 3: fully adjusted sex, age, ethnicity, poverty level, smoke, alcohol user, diabetes, hypertension, BMI, CVD, antineoplastic drug user, and neoplastic type. Bold values indicate statistically significance of *p* < 0.05.

Abbreviations: BMI, body mass index; CKD, chronic kidney disease; CVD, cardiovascular disease; HR, hazard ratio.

## 4 DISCUSSION

Cancer and CVD are the two leading causes of morbidity and mortality in the US population. These two seemingly unrelated diseases are intertwined, thus, the blossoming field of cardio‐oncology has been rapidly paid attention to the world. CS has dramatically increased in the past decades, and these survivors may be more likely to increase the incidence of CVD and die from CVD.^14^ Study reported that metabolism is a critical mechanism in both cancer and CVD.^15^ Cardiomyocytes share many common metabolic pathways with cancer cells, and chemotherapy targets metabolic vulnerabilities in cancer, suggesting that metabolic reprogramming may adversely impact the heart. Based on current knowledge, CVD and cancer share some common risk factors, such as diabetes, obesity, and dyslipidemia.^16^, ^17^

Except for the above risk factors, elevated SUA levels also contributed to disturbing the normal metabolic pathway. High levels of SUA (hyperuricemia) are associated with obesity, diabetes, inflammation, and metabolic syndrome, suggesting that it can act as a metabolic regulator and in the pathogenesis of various diseases including CVD and cancer.^18^, ^19^ Nonetheless, epidemiological studies evaluating the levels of SUA and CVD‐related risk in the CS population remain sparse. To our best knowledge, this is the first study to explore the potential association between hyperuricemia and the incidence of cardiovascular events among the CS population. In this cross‐sectional and longitudinal study, the main findings are as follows: (1) CS participants with hyperuricemia were significantly increased the risk of CVD. The relationship between SUA levels and CVD followed a nonlinear pattern, with the risk of CVD increasing when SUA levels exceeded physiological concentrations; (2) in CS population, although the levels of SUA had a weak positive correlation with all‐cause of mortality and CVD mortality, the fully adjusted model showed that there was no such association. (3) Furthermore, our study also indicated that the SUA levels were negatively associated with CFR. Therefore, we further investigated whether the elevated mortality rate in CS was directly associated with higher SUA levels, or if SUA contributed indirectly to increased mortality by coexisting with renal dysfunction. Subgroup analysis indicated that CS with CKD was more sensitive to the SUA levels, although this did not reach statistical significance in the fully adjusted model. While there was no association between hyperuricemia and the all‐cause and CVD mortality in CS participants without CKD.

SUA is the end‐product of purine metabolism, and the normal SUA levels are usually between 170 and 360 μmol/L. Previous knowledge thought that UA was an independent risk factor for all‐cause and CVD mortality in the general population, but not all observational studies agreed with this viewpoint.^20^, ^21^, ^22^, ^23^, ^24^, ^25^ Therefore, most authoritative guides have not made a recommendation on whether hyperuricemia is a risk factor for CVD.^26^ In addition, there was no such clinical study focused on the CS population. In this study, we indicated that hyperuricemia was an independent risk factor for the incidence of CVD after adjusting overall potential confounding covariates. Study used RCS method further identified that the SUA levels ≥345 μmol/L increased CVD incidence in CS, because SUA was a powerful antioxidant at physiological concentrations, while it could act as a pro‐oxidant molecule at high levels.^27^ However, the association between hyperuricemia and mortality was abolished after further adjusting metabolic‐relative factors, such as CKD. Interestingly, our results suggested that hyperuricemia was positively associated with the all‐cause and CVD mortality in CS participants with CKD in model 2, while, there was no such correlation in CS participants without CKD, as CKD might be an intermediate variable that mediated the effects of hyperuricemia on CVD and death. It is acknowledged that approximately two‐thirds of UA are excreted through kidneys, and the relationship between hyperuricemia and kidney function has been proved, and both of them could drive to CVD and mortality.^28^

This exploratory analysis has provided valuable insights into potential risks of CVD incidence and mortality in the CS population who had hyperuricemia. However, this study also has some limitations. First, we did not further study the impact of urate‐lowering agents on outcomes due to incomplete medication information. Second, we only used GFR < 60 mL/min/1.732 m^2^ to definite the CKD, and did not further classify the stages of CKD based on the GFR. Third, the association between SUA levels and mortality is complex, further study is needed to identify if CKD is an intermediate variable that affects the relationship between SUA and mortality in CS.

## 5 CONCLUSION

This study was the first to analyze the association between SUA and CVD incidence and mortality (all‐cause/CVD mortality) in the CS population. In CS with hyperuricemia, the incidence of CVD might increase. In addition, hyperuricemia with CKD in CS was associated with higher all‐cause and CVD‐related mortality, although this did not reach statistical significance in the fully adjusted model. In summary, this study might offer novel insights and arguments for the field of cardio‐oncology research, which may provide a possible pathophysiology of hyperuricemia and CVD mortality in CS.

## 6 PERSPECTIVES

### 6.1 Competency in medical knowledge

Uric acid significantly increases the risk of cardiovascular diseases and CKD, leading to elevated all‐cause mortality and CVD mortality in the general population.

### 6.2 Translational outlook

These findings suggested that hyperuricemia had higher all‐cause and CVD‐related mortality in the CS population, especially in CS with hyperuricemia and CKD, which could provide novel insights and arguments for cardio‐oncology research.

## AUTHOR CONTRIBUTIONS

**Yanlin Chen:** Data curation (equal); formal analysis (equal); investigation (equal); methodology (equal); validation (equal); writing – original draft (equal); writing – review and editing (equal). **Yuhan Chen:** Formal analysis (equal); investigation (equal); resources (equal); supervision (equal); validation (equal); visualization (equal). **Weidong Lin:** Conceptualization (equal); investigation (equal); supervision (equal). **Lu Fu:** Methodology (equal); visualization (equal). **Huiyi Liu:** Formal analysis (equal); validation (equal). **Sijia Pu:** Conceptualization (equal); software (equal). **Haowei Chen:** Methodology (equal); visualization (equal). **Hong Yi:** Investigation (equal); writing – original draft (equal). **Yumei Xue:** Conceptualization (equal); funding acquisition (equal); project administration (equal); supervision (equal); writing – review and editing (equal).

## FUNDING INFORMATION

This work was supported by the Science and Technology Programs of Guangdong Province (2020B1111170011 and 2023B110009).

## CONFLICT OF INTEREST STATEMENT

The authors have reported that they have no relationships relevant to the contents of this paper to disclose.

## DATA AVAILABLITY STATEMENT

The datasets used in this manuscript are publicly available from the NHANES website: https://www.cdc.gov/nchs/nhanes/index.htm.

## Supporting information


Data S1:
