# Supplementary material for: Impact of hyperuricemia and chronic kidney disease on the prevalence and mortality of cardiovascular disease in cancer survivors
Source: Cancer Med. 2024 Apr 30;13(9):e7180. doi: 10.1002/cam4.7180 (PMC11058684; doi:10.1002/cam4.7180)
Supplement: Supplementary file 1 — Data S1: [file CAM4-13-e7180-s001.docx]

| **System** | **Cancer Type** | **Numbers of Cancer Survivors** | | |
| --- | --- | --- | --- | --- |
|  |  | **Alive** | **Death** | **Total** |
| Gastrointestinal Neoplasm | Esophagus (esophageal) | 7 | 10 | 17 |
|  | Liver | 7 | 6 | 13 |
|  | Mouth/tongue/lip | 10 | 9 | 19 |
|  | Pancreas (pancreatic) | 5 | 2 | 7 |
|  | Rectum (rectal) | 8 | 2 | 10 |
|  | Stomach | 15 | 7 | 22 |
|  | Colon | 120 | 79 | 199 |
| Endocrine System Tumor | Breast | 373 | 127 | 500 |
|  | Thyroid | 63 | 7 | 70 |
| Genitourinary System Tumors | Bladder | 47 | 32 | 79 |
|  | Cervix (cervical) | 210 | 18 | 228 |
|  | Gallbladder | 2 | 0 | 2 |
|  | Kidney | 43 | 17 | 60 |
|  | Ovary (ovarian) | 61 | 9 | 70 |
|  | Prostate | 338 | 161 | 499 |
|  | Testis (testicular) | 14 | 2 | 16 |
|  | Uterus (uterine) | 105 | 20 | 126 |
| Circulatory System Neoplasm | Blood | 3 | 4 | 7 |
|  | Lymphoma/Hodgkin's disease | 51 | 18 | 69 |
|  | Leukemia | 24 | 7 | 31 |
| Respiratory System Neoplasm | Larynx/ windpipe | 7 | 4 | 11 |
|  | Lung | 38 | 33 | 71 |
| Neurological Neoplasms | Brain | 10 | 4 | 14 |
|  | Nervous system | 1 | 0 | 1 |
|  | Bone | 5 | 5 | 10 |
|  | Skin (non-melanoma) | 393 | 126 | 519 |
|  | Skin (don't know what kind) | 170 | 75 | 245 |
|  | Melanoma | 153 | 46 | 199 |
|  | Soft tissue (muscle or fat) | 4 | 1 | 5 |
|  | Other | 114 | 43 | 157 |

Table 1. Numbers of Cancer Survivors by Cancer Type and Survival Status, NHANES 2007-2015

| **Outcomes** | **β(95% CI)** | **P value** |
| --- | --- | --- |
| Uric acid~GFR |  |  |
| Model 1 | -1.58(-1.75, -1.41) | **<0.0001** |
| Model 2 | -1.83(-2.06, -1.60) | **<0.0001** |
| Model 3 | -1.97(-2.42, -1.52) | **<0.0001** |

Table 2. Weighted linear regression models show a negative association between the level of uric acid and GFR. Model 1: no covariate was adjusted. Model 2: adjusted for sex, age, and ethnicity. Model 3: fully adjusted sex, age, ethnicity, poverty level, smoke, alcohol user, diabetes, hypertension, BMI, CKD, antineoplastic drug user, and neoplastic type.
